# How Sodium and Calcium Ions Pass Through Batrachotoxin-Bound Sodium Channel

**DOI:** 10.3390/toxins17100520

**Published:** 2025-10-21

**Authors:** Boris S. Zhorov

**Affiliations:** 1 Department of Biochemistry and Biomedical Sciences, Master University, Hamilton, ON L8S 4K1, Canada; zhorov@mcmaster.ca; 2 I.M. Sechenov Institute of Evolutionary Physiology and Biochemistry, Russian Academy of Sciences, St. Petersburg 194223, Russia

**Keywords:** molecular modeling, Monte Carlo energy minimizations, ion selectivity of sodium channels, protonatable nitrogen atoms

## Abstract

Steroidal sodium channel agonist batrachotoxin (BTX), one of the most potent animal toxins, dramatically increases calcium permeation and alters other channel characteristics. In a cryoEM structure of rat sodium channel Nav1.5 with two BTX-B molecules, one toxin binds between repeats III and IV and exposes to the pore lumen two oxygen atoms and protonatable nitrogen. The mechanism of ion permeation and selectivity in BTX-bound channel is unclear. Here Monte Carlo energy-minimized profiles of sodium and calcium ions pulled through the pore were computed in models with various protonated states of the DEKA lysine and BTX-B. The only model where the ions readily passed by the DEKA lysine and BTX-B involved their deprotonated nitrogens. In this model, electronegative atoms of BTX-B attracted a permeant cation that stabilized the “dunked” lysine through electrostatic interactions and nearby water molecules. This would retard reprotonation of the lysine and its “uplifting” to the DEKA carboxylates, which otherwise attracts calcium. The results suggest how sodium and calcium ions pass through BTX-bound sodium channel and why BTX increases calcium permeation. The study supports an earlier hypothesis that during the sodium ion permeation cycle, the DEKA lysine alternates between uplifted and dunked conformations in the protonated and deprotonated states, respectively, while the sodium-displaced proton and the sodium ion nullify the net electrical charge at the DEKA region.

## 1. Introduction

Batrachotoxin (BTX), one of the most powerful animal toxins [1,2,3,4], is a steroidal sodium-channel agonist first isolated from the skin of Colombian frog *Phyllobates bicolor* [5]. BTX binds preferentially to the open voltage-gated sodium channels [6,7,8], shifts the voltage-dependence of activation in the hyperpolarizing direction, inhibits inactivation, and reduces selectivity to sodium ions and the channel conductance [9,10,11,12]. Intriguingly, BTX increases manifold the channel permeability for Rb^+^, Cs^+^, and Ca^2+^ [8]. Due to its high affinity and specificity to sodium channels, BTX is a useful tool to explore their electrophysiological properties.

The pore-forming alpha-subunit of Nav1.x channels folds from a single polypeptide chain of four homologous, but not identical, repeats. In each repeat, transmembrane (TM) helices S1–S4 form a voltage-sensing domain, while helices S5, S6, and P-loop with membrane-descending helix P1 and membrane-ascending helix P2 contribute a quarter to the pore domain. The ion permeation pathway has two parts: the outer pore exposed to the extracellular space and the inner pore, which in the open channel is exposed to the cytoplasm. The outer pore contains the selectivity filter residues Asp, Glu, Lys, and Ala (the DEKA ring), outer carboxylates, and two rings of the pore-exposed backbone carbonyls below the DEKA ring. The outer pore is targeted by various toxins, including tetrodotoxin and saxitoxin. The inner pore has binding sites for many small-molecule drugs and some toxins; for reviews see [13,14].

Multiple studies addressed the mechanism of BTX action at the location of its binding site. In earlier studies, BTX was proposed to bind between the lipid membrane and the channel and allosterically activate it. Ging-Kuo Wang and coauthors demonstrated that BTX-sensing residues are located in all the four S6 helices [15,16,17,18], implying that BTX binds inside the pore, and raising a question why the bulky toxin does not block the current. The question was addressed in a homology model of a BTX-bound sodium channel Nav1.4 [19] based on the crystal structure of open prokaryotic potassium channel MthK [20]. In this model, the sodium ion was proposed to move between oxygen atoms of BTX and the pore-lining channel residues. The model directed mutational studies of the Nav1.4 channel, which revealed three pore-facing residues whose lysine substitutions affected the BTX action [21,22,23]. A study of a cockroach sodium channel identified three additional residues whose alanine substitutions decreased the BTX action [24]. Intriguingly, BTX was also shown to modify homotetrameric prokaryotic sodium channels NaChBac and NavSp1, and mutations of the pore-facing residues in these channels affected the BTX action [25].

CryoEM structures of eukaryotic sodium channels greatly advanced understanding of their structure, pharmacology, and toxicology. In these structures, the selectivity filter DEKA lysine (Lys) in the “dunked” conformation approaches two rings of the pore-facing backbone carbonyls, which form a cation-attractive exit from the outer pore. However, the mechanism of ion selectivity and ion permeation remains the matter of controversy [13,14]. Molecular dynamic studies [26,27,28] agree that Na^+^ permeation requires protonated lysine (Lys^+^). An alternative hypothesis proposes that “uplifted” Lys^+^ is salt-bridged to the DEKA glutamate (Glu^−^); an incoming Na^+^ ion displaces H^+^ from Lys^+^, deprotonated Lys^0^ escorts Na^+^ to the outer pore exit where Na^+^-displaced H^+^ rejoins Lys^0^, and Lys^+^ uplifts to Glu^−^ to complete the permeation cycle [29]. Both theories suggest a zero net charge in the Na^+^-occupied DEKA region.

Recently, a cryoEM structure (PDB ID: 86tl) of inactivated rat cardiac sodium channel rNav1.5 with two molecules of BTX-B was published [4]. BTX-B is a close structural analog of BTX and has the same action in sodium channels as BTX [4]. Therefore, in this study, the abbreviation BTX also refers to BTX-B. In the cryoEM structure, one BTX molecule binds deeply in the interface between repeats I and IV, far from the pore lumen. The second BTX molecule binds in the interface between repeats III and IV and exposes to the pore lumen its benzoate moiety, two methyl groups, and protonatable nitrogen. The cryoEM structure did not elucidate the protonation states of BTX and the DEKA lysine. These are major unknowns, which are critical for understanding how Na^+^ permeates through BTX-bound channel and why BTX dramatically changes the ion selectivity.

Here, Monte Carlo energy-minimized energy profiles of Na^+^ and Ca^2+^ ions were computed in four models of BTX-bound rNav1.5 channel with Lys^+^ or Lys^0^ and BTX^+^ or BTX^0^. Only in the model with Lys^0^/BTX^0^, Na^+^ and Ca^2+^ ions favorably interacted with the pore-exposed electronegative atoms of BTX in the III/IV repeat interface. In this model, a BTX^0^-bound permeant cation would stabilize the dunked Lys^0^ via electrostatic interactions and H-bonds with nearby water molecules, retard its reprotonation and uplifting to Glu^−^ [29]. This scenario may explain the increased calcium permeability of the channel [8], a paradoxical effect of BTX that does not bind to the selectivity filter.

## 2. Results

The pore domain from the cryoEM structure of rNav1.5 channel with two BTX molecules (8tl6) [4] was used as a starting point in the computations. The structure was downloaded from the database of P-Loop Ion Channels (PLICs) (www.plic3da.com), accessed on 22 September 2025, where hundreds of crystal and cryoEM structures of P-loop ion channels with universal residue labels are 3D aligned in such a way that their pore axis coincides with the *z*-axis of the Cartesian system coordinates [30]. A PLIC label refers to the channel repeat, segment in the repeat, and residue position relative to the reference residue, which is most conserved in the segment. The reference residues have labels *rs*.550 in TM helices and *r*5.850 in P-loops, where *r* stands for repeat (A to D) and *s* stands for the TM segment (1 to 6). Repeats A–D are arranged clockwise when viewed from the extracellular side. For example, the selectivity filter DEKA lysine in position 1421 of the rNav1.5 channel is designated K^C5.850^ in this channel and in all other eukaryotic Na^+^ channels. Relations between the UniProt numbers and PLIC labels of residues in the pore domain of the rNav1.5 channels are given in Table 1.

### 2.1. Model with BTX^0^/Lys^0^

**Figure 1:**
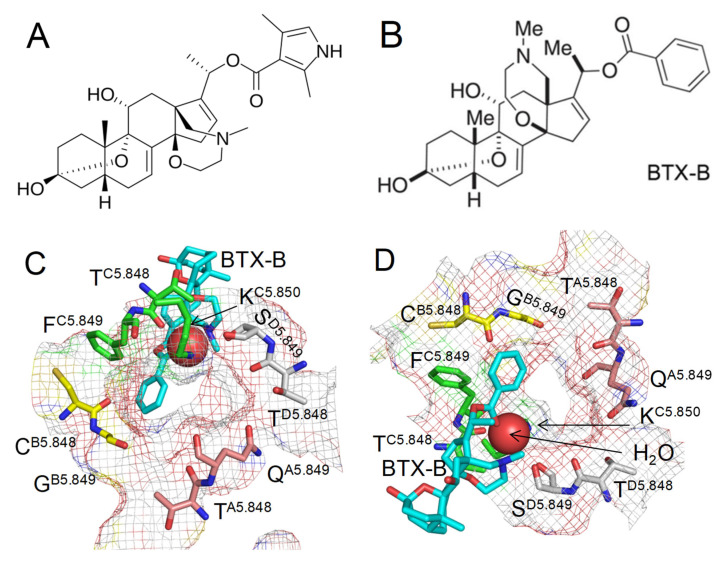
Narrow exit from the outer pore to the inner pore in the cryoEM structure of BTX-bound channel (8t6l). (**A**,**B**) Chemical structures of BTX and BTX-B. The only difference is the pyrrole ring in BTX and the phenyl ring in BTX-B. (**C**,**D**) Extracellular (**C**) and intracellular (**D**) views at BTX-B in the cryoEM structure. Backbone and sidechain atoms are shown for all the residues except K^C5.850^, for which only the sidechain is shown. Carbon atoms in repeats I, III, III, and IV are magenta, yellow, green, and gray, respectively. BTX carbons are cyan. A red sphere is a water molecule. Mesh rendering shows that the region is crowded, but a Na^+^ ion may pass escorted by one or two water molecules ahead and/or behind it.

In the cryoEM structure 8t6l, a water molecule is seen 3.4 Å from the sidechain nitrogen of the DEKA lysine and 3.7 Å from the BTX nitrogen. In a cryoEM structure with resolution ~3 Å, it is hardly possible to distinguish a water molecule from a Na^+^ ion [31], and respective electron density could be a Na^+^ ion. Both possibilities are explored in this study. BTX crosses the lipid membrane in the unprotonated state. In the cryoEM structure, the protonation states of BTX and the DEKA lysine are unknown. BTX partially occludes the upper part of the inner pore, leaving a narrow entrance for a Na^+^ ion from the outer pore (Figure 1B,C). The Na^+^ ion may interact with the water molecule shown in the cryoEM structure. Alternatively, the Na^+^ ion could replace the water molecule. The latter possibility would strongly depend on the protonation state of BTX and the DEKA lysine. To explore various possibilities, four models were constructed with the following configurations of BTX and Lys: Lys^0^/BTX^0^, Lys^+^/BTX^0^, Lys^0^/BTX^+^, and Lys^+^/BTX^+^. Each model was filled with explicit water molecules, as described in Methods.

**Figure 2:**
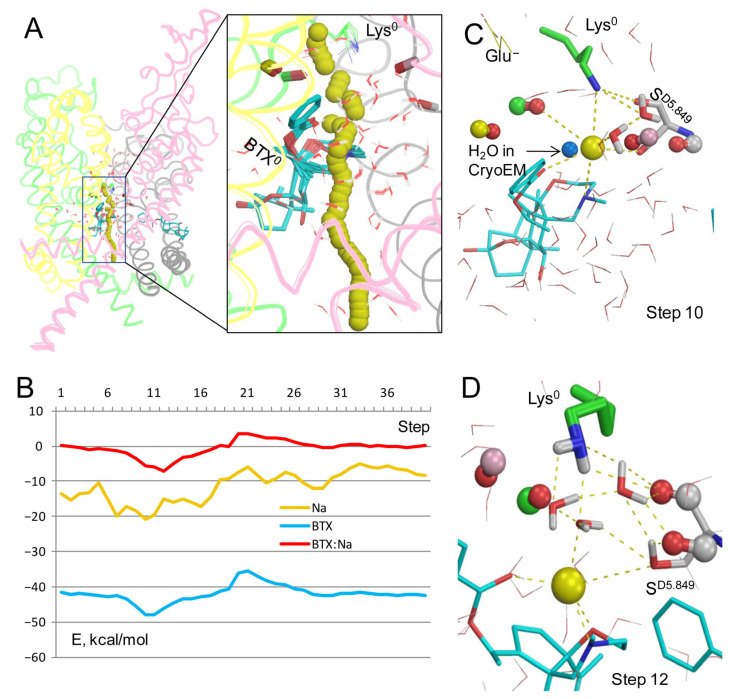
Na^+^ trajectory in model BTX^0^/Lys^0^ with explicit water molecules. (**A**) Superposition of 40 MC-minimized structures. Repeats A, B, C, and D are pink, yellow, green, and gray, respectively. Backbone carbonyls in positions 5.848 are shown by sticks. (**B**) Energy profiles of Na^+^, BTX^0^, and Na^+^: BTX^0^ interactions. All profiles have minima at step 12. (**C**) Na^+^ contacts at step 10. Backbone carbonyls in positions 5.848 are shown as “dumbbells”, with red oxygens and carbons colored as respective backbones in panel (**A**). Na^+^ is ~3.3 Å from the nitrogen atoms in BTX^0^ and Lys^0^ and within 4 Å from four oxygen atoms (BTX, T^C5.848^, S^D5.849^, and a water molecule). Blue sphere is a water molecule in the cryoEM structure. (**D**) At step 12, Na^+^ is coordinated by three polar atoms of BTX and two water molecules. The NH_2_ group of Lys^0^ forms H-bonds with the backbone carbonyl of S^D5.849^ and three water molecules, which are engaged in a network of H-bonds.

In the model with BTX^0^/Lys^0^, Na^+^ closely approached BTX (Figure 2A). The energy of Na^+^ is minimal at steps 8 to 14 (Figure 2B) due to attraction to electronegative atoms of BTX. Position of Na^+^ at step 10 is very close to that of the water molecule in the cryoEM structure 8t6l (Figure 2C). At this step, Na^+^ is equidistant (~3 Å) from the nitrogen atoms in BTX^0^ and Lys^0^ and it favorably interacts with three electronegative atoms of BTX, two water molecules, sidechains of Lys^0^ and S^D5.849^, and the backbone carbonyls of T^C5.848^ and S^D5.849^. At step 12, Na^+^ is chelated by three electronegative atoms of BTX^0^ and favorably interacts with a water molecule and the sidechain of S^D5.849^ (Figure 2D). It should be noted that since the entropy is not computed, the energy profiles reflect enthalpy rather than free energy. Due to this limitation the Na^+^ energy is negative all along the inner pore, in spite of the cryoEM structure 8t6l capturing the channel in an impermeable inactivated state. The energy is maximal at steps 33–35 around the activation gate. Another maximum of Na^+^ energy is seen at step 21, where energies of BTX^0^ and BTX^0^: Na^+^ interactions are also maximal (Figure 2B). Importantly, stabilization of Na^+^ by BTX^0^ and related stabilization of the dunked conformation of Lys^0^ (Figure 2C,D) may explain why BTX dramatically increases calcium permeability [8], as discussed in a later section.

### 2.2. Model with BTX^0^/Lys^0^ and a Water Molecule from the cryoEM Structure

**Figure 3:**
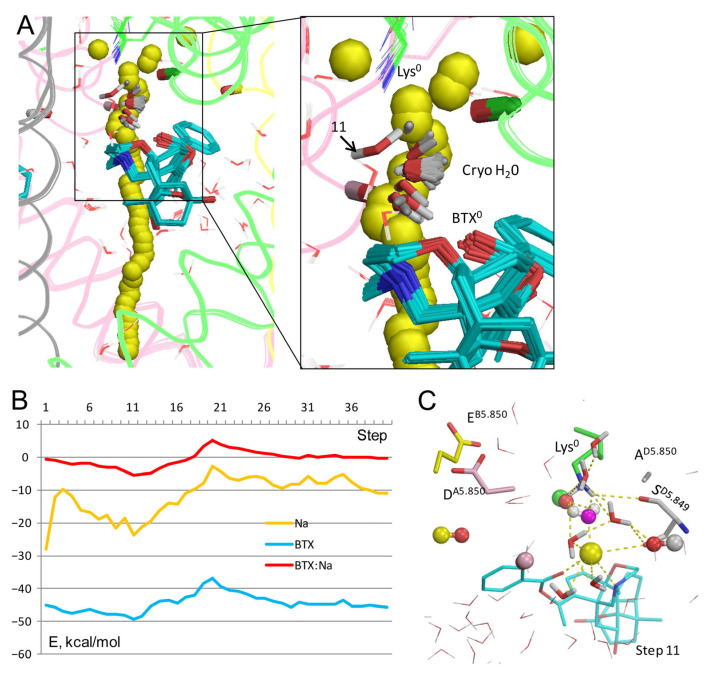
Na^+^ trajectory in model BTX^0^/Lys^0^/cryo-H_2_0**.** (**A**) Superposition of 40 MC-minimized structures. Heavy atoms of BTX^0^ molecules and the water molecule in structure 8t6l (sticks) were pinned to experimental positions. However, at step 11, the Na^+^ ion clashed with the cryoEM-H_2_O molecule, which, unlike molecules from the water sphere, did not disappear upon the clash, but shifted despite the pin penalty energy, interacted with the Na+ sodium ion, and formed several H-bonds, shown in panel (**C**). (**B**) All energy profiles have minima at step 11. Na^+^ energy has a maximal value at step 20 due to repulsion from BTX, but the energy is still negative, implying that the current would permeate. (**C**) MC-minimized structure at step 11. The DEKA residues are shown by sticks, and backbone carbonyls in position 5.850 as dumbbells. Na^+^ has displaced the cryoEM water molecule (spheres with magenta oxygen) and engaged in favorable contacts with two electronegative atoms of BTX^0^, four water molecules, and S^D5.849^.

In the cryoEM structure 8t6l, the electron density of a single atom between BTX and the dunked lysine (HOH A3101 in the PDB file) is interpreted as a water molecule. Figure 2C shows this molecule (cryoEM-H_2_O) would be close to a Na^+^ ion between Lys^0^ and BTX^0^. To explore how Na^+^ may move through the channel with the explicit cryoEM-H_2_O molecule, a model BTX^0^/Lys^0^ was constructed with the cryoEM-H_2_O molecule treated as a ligand, and the experimental position of its oxygen was biased by a pin constraint. This was necessary to exclude a possibility that the cryoEM-H_2_O molecule would “disappear” upon collision with the Na^+^ ion, as do molecules from the water sphere (see Methods). The Na^+^ ion trajectory (Figure 3A) was similar to that in Figure 2A,B. The cryoEM-H_2_O molecule fluctuated around its experimental position (Figure 3A).

However, upon collusion with the Na^+^ ion at step 11, the cryoEM-H_2_O molecule moved from the experimental position despite the penalty energy; this position is marked “11” in the Figure 3A enlargement. The displaced cryoEM-H_2_O molecule was engaged in multiple favorable contacts with the Na^+^ ion, nearby water molecules, and the dunked Lys^0^. The latter was stabilized by the displaced cryoEM-H_2_O molecule, water molecules from the water sphere, the sidechain of S^D5.849^, the backbone carbonyl of T^C5.848^, and the Na^+^ ion chelated by BTX^0^ (Figure 3C). Thus, the BTX^0^-chelated Na^+^ ion in its water environment stabilized the dunked Lys^0^, regardless of whether the electron density in the cryoEM structure is interpreted as a Na^+^ ion (Figure 2C,D) or a water molecule (Figure 3C).

### 2.3. Model of the Apo-Channel with Lys^0^

**Figure 4:**
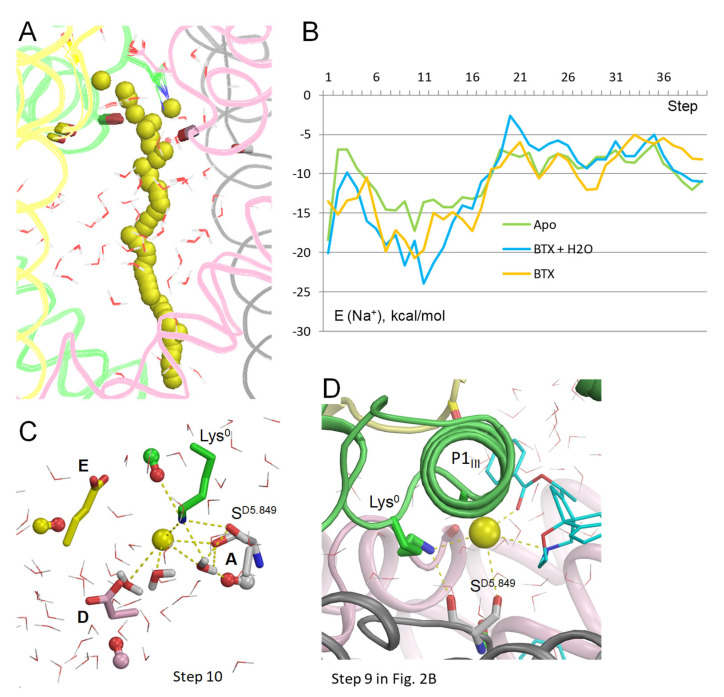
Na^+^ in apo-channel model with Lys^0^. (**A**) Trajectory of Na^+^. (**B**) Energy of Na^+^ in three models: no BTX (apo), BTX^0^, and BTX^0^/ryoEM-H_2_0. In all the models, Na^+^ in the inner pore has low energies in steps 6–16. In the models with BTX, Na^+^ energy has a barrier at step 20 due to repulsion from BTX. (**C**) At the lowest energy step 10 in the apo-channel, Na^+^ is coordinated by three water molecules, Lys^0^, and S^D5.849^. (**D**) View at step 9 (Figure 2B) in model BTX^0^/Lys^0^ along helix P1_III_. Besides direct contacts with BTX, S^D5.849^, Lys^0^, and OC_T^C5.848^, Na^+^ is stabilized by electrostatic interaction with dipole of helix P1_III_.

Na^+^ trajectory in the model without BTX (Figure 4A,B) is rather similar to the models with Lys^0^/BTX^0^. The Na^+^ energy has a sharp minimum at step 10 due to favorable contacts with three water molecules, S^D5.849^ and Lys^0^ (Figure 4C). However, this minimum is considerably shallower than analogous minima in models with BTX^0^/Lys^0^ and BTX^0^/Lys^0^/cryoEM-H_2_O. In all three models, Na^+^ at steps 5–17 is stabilized by the above-described contacts, as well as by macrodipoles in P1 helices [32], specifically, with helix P1_III_ in this channel (Figure 4D).

Thus, Na^+^ trajectories in the apo-channel model and models with BTX^0^ consensually predict a preferable Na^+^ energy in the central cavity and higher energy after step 17. As compared to the apo-channel, BTX^0^ further stabilized Na^+^ in the central cavity, but imposed an energy barrier at steps 20–25. These results suggest that Na^+^ would pass through BTX-bound channel, although with a lower single-channel conductance than in unmodified channels, as demonstrated earlier [12]. Importantly, additional stabilization of Na^+^ at steps 9–12 in models with BTX^0^ would also stabilize the dunked conformation of Lys^0^, whose amino group interacts with the Na^+^ ion directly and through nearby water molecules.

### 2.4. Models with BTX^0^/Lys^0^ and Implicit Solvent

**Figure 5:**
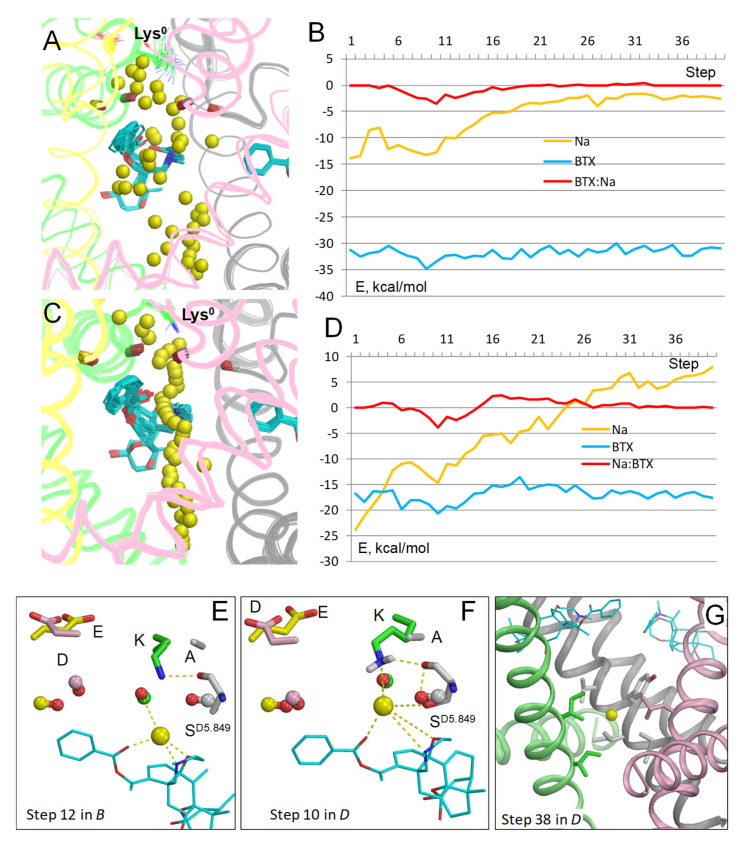
Na^+^ trajectories in model BTX^0^/Lys^0^ without explicit water molecules. (**A**) Na^+^ trajectory with electrostatic interactions computed using distance- and environment-dependent dielectric function [33]. The DEKA residues are thin lines, backbone carbonyls at positions 5.848 are sticks. Na^+^ significantly deviated from the pore axis at many steps. (**B**) Energy profiles in the trajectory shown in panel (**A**). Position of the BTX:Na^+^ energy minimum (steps 9–13) is similar to that in model with explicit waters (Figure 2B). (**C**) Na^+^ trajectory computed with implicit solvent [34]. (**D**) Energy profiles from the trajectory shown in panel (**C**). Note a positive energy of Na^+^ at the intracellular part of the pore. (**E**) Close-up view of step 12 from panel (**B**). Na^+^ is chelated by three polar atoms of BTX^0^, while Lys^0^ adopts a dunked conformation that was not biased in computations (note diverse conformations of Lys^0^ in panel (**A**). (**F**) Close-up view of step 10 from panel (**D**). Na^+^ is chelated by three polar atoms of BTX^0^. (**G**) Close-up view of step 38 in panel (**D**). Na^+^ has a positive energy due to hydrophobic environment at the closed activation gate in the inactivated channel.

To test sustainability of the above conclusion to other computational methods, configuration BTX^0^/Lys^0^ was explored in two additional models. In the first model, electrostatic interactions were calculated with the environment- and distance-dependent dielectric function [33]. Na^+^ significantly deviated from the pore axis at many levels of the pore (Figure 5A), unlike in the model with explicit water molecules whose mobility is limited in MC-minimizations. In this model, the sidechain of Lys^0^, which was not biased to the experimental structure, fluctuated, and its NH_2_ group approached Na^+^ at the exit from the outer pore (Figure 5A). The energy profiles of Na^+^ and Na^+^: BTX^0^ interactions have minima at steps 10 to 15 (Figure 5B), as in the model with explicit water molecules (Figure 2B). In step 12, Na^+^ was chelated by three electronegative atoms of BTX^0^ and was attracted by the backbone carbonyl of T^C5.848^ (Figure 5E).

In computations with the implicit solvent [34], Na^+^ also closely approached BTX^0^ (Figure 5C). The Na^+^ trajectory jagged less that in computations with the environment- and distance-dependent dielectric function (Figure 5A), but more than in the model with explicit waters (Figure 2A). The energy minima for Na^+^, BTX^0^, and BTX^0^:Na^+^ interactions are seen at step 10 (Figure 5D), which is 1 Å above the energy minima at step 12 in the model with explicit water molecules (Figure 2B). At step 10, Na^+^ was chelated by three electronegative atoms of BTX^0^ (Figure 5F). An important feature of this model is a positive energy of Na^+^ at the cytoplasmic end of the pore where Na^+^ is surrounded by hydrophobic groups of the closed activation gate (Figure 5G). 

Thus, the three methods, which consider the aqueous environment within the pore in models with configuration BTX^0^/Lys^0^, unambiguously predicted that a Na^+^ ion, which enters the inner pore, would be attracted by three electronegative atoms of BTX^0^. An important advantage of the method with explicit water molecules are structures with a Na^+^ ion coordinated at the same pore level where a water molecule is seen in the cryoEM structure (Figure 2C), implying stabilization of the dunked Lys^0^ by the Na^+^ ion and nearby water molecules. A disadvantage is the negative energy of Na^+^ at the closed activation gate due to electrostatic interactions with water molecules above and beyond the ion. In the models with the environment- and distance-dependent dielectric function [33] and implicit water molecules [34], a water molecule would fill the void between BTX^0^-chelated Na^+^ ion and the dunked Lys^0^ (Figure 5E,F) and stabilize the latter as in the models with explicit waters. In the above models, energy of Na^+^ at the activation gate (Figure 5G) either approached zero (Figure 5B) or was positive (Figure 5D), indicating that these methods are sensitive to the hydrophobic environment of Na^+^ at the activation gate. Since the goal of this study was to explore how a Na^+^ ion would pass by BTX in models with different protonated states of the DEKA lysine and BTX, the advantage of the model with explicit waters is more important that its disadvantage. Therefore, models with protonated DEKA lysine and BTX were explored using explicit water molecules.

### 2.5. Model with BTX^0^/Lys^+^

**Figure 6:**
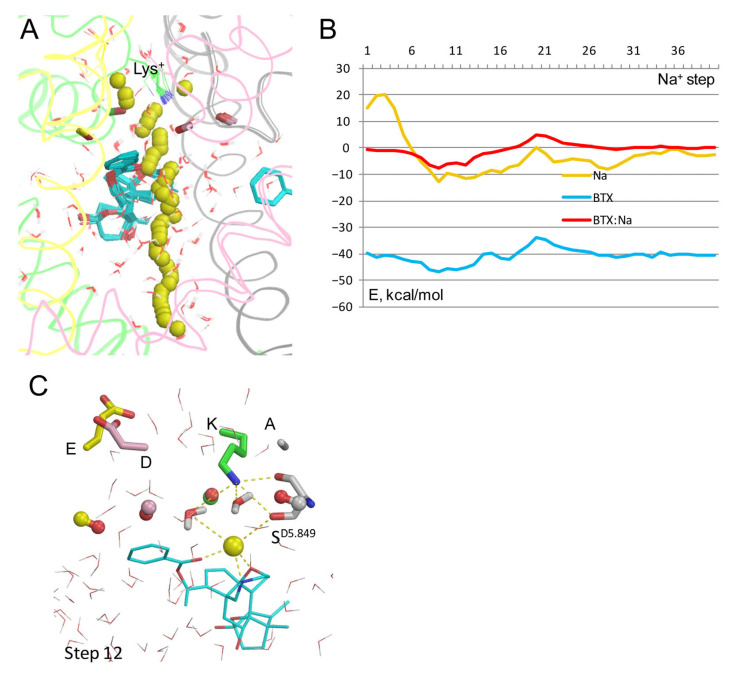
Na^+^ trajectory in model with Lys^+^/BTX^0^ and explicit water molecules. (**A**) Na^+^ trajectory. Lys^+^ conformation is biased to the cryoEM structure by pins that prevented uplifting of Lys^+^ to the DEKA glutamate. (**B**) Energy profiles. Na^+^ energy has a maximum at step 3 due to electrostatic repulsion from Lys^+^. Such energy barrier would retard Na^+^ leaving from the outer pore. The BTX^0^: Na^+^ energy is minimal at step 12, where the ion is attracted to BTX^0^ and maximal at step 19. (**C**) Membrane view of step 12. Backbone carbonyls in positions 5.848 are shown as dumbbells. Lys^+^ donates two H-bonds to D^5.849^ and repels Na^+^, which is chelated by BTX^0^ as in other models.

The energy profile of Na^+^ in the model with Lys^+^/BTX^0^ has a maximum at steps 2–3 due to electrostatic repulsion from Lys^+^ whose sidechain was pinned to the experimental position (Figure 6A,B). Energy of Na^+^ is negative at steps 7–17, but the wide energy minimum at these steps is shallower than analogous minima in models with BTX^0^/Lys^0^ (Figure 4B). In step 12, Na^+^ was chelated by three electronegative atoms of BTX^0^, two water molecules, and the backbone carbonyl of S^D5.849^, while Lys^+^ donated H-bonds to the backbone and sidechain oxygens of S^D5.849^ and two water molecules (Figure 6C). The energy barrier of Na^+^: BTX^0^ interactions at step 20 is higher than that in models with Lys^0^/BTX^0^ (Figure 4B).

Thus, the energy profile of Na^+^ in the model with BTX^0^/Lys^+^ has two distinguishing features vs. model with BTX^0^/Lys^0^: (i) a high energy barrier as Na^+^ passes by Lys^+^ and (ii) higher energy at steps 7–40, indicating that Na^+^ experiences electrostatic repulsion from Lys^+^ all along the inner pore. The dunked Lys^+^ opposes Na^+^ movement at the exit from the outer pore, but does not prevent Na^+^ movement against BTX^0^ and its chelation by the toxin. At the low energy step 12, Na^+^ would destabilize the dunked Lys^+^ in contrast to the models with BTX^0^/Lys^0^ where Na^+^ stabilized the dunked Lys^0^. This difference is important in view of the dramatic change in the ion selectivity of BTX-modified channels [8] as discussed in a later section.

### 2.6. Models with BTX^+^/Lys^0^ and BTX^+^/Lys^+^

**Figure 7:**
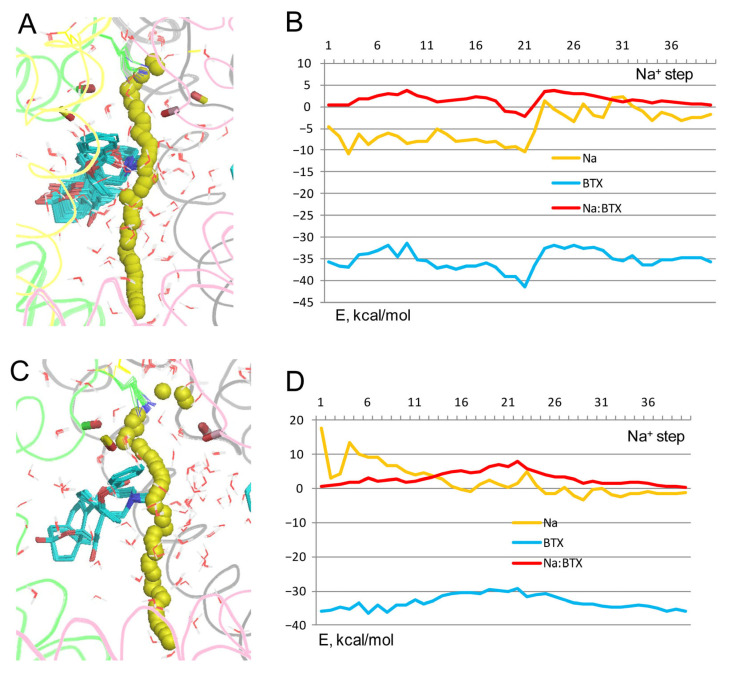
Na^+^ in models with BTX^+^. (**A**) Trajectory of Na^+^ in model Lys^0^/BTX^+^. Na^+^ is attracted to Lys^0^ at step 3 and avoids the protonated nitrogen of BTX^+^. (**B**) Energy profiles from trajectory (**A**). BTX^+^:Na^+^ energy varies from +3.7 kcal/mol at step 7 due to electrostatic repulsion to −2.3 kcal/mol at step 21. (**C**) Trajectory of Na^+^ in model Lys^+^/BTX^+^. Repeat B is removed for clarity. Na^+^ experiences repulsion from Lys^+^ at steps 1 and 4 and avoids BTX^+^. (**D**) Energy profiles from trajectory (**C**). The BTX^+^:Na^+^ energy is positive all along the inner pore. It has a maximum at step 22, where the ion experiences repulsion from the hydrophobic groups of BTX^+^.

In the model with BTX^+^/Lys^0^, Na^+^ experienced repulsion from BTX^+^ all along the trajectory, except for steps 19–22 where the BTX^+^: Na^+^ energy was weakly negative due to nonbonded attraction of Na^+^ by the toxin (Figure 7A,B). However, Na^+^ has a negative energy at steps 1–20 due to its favorable hydration. And unlike in models with BTX^0^/Lys^0^ (Figure 4B), the Na^+^ energy beyond step 23 fluctuated around zero and had small positive values despite the presence of explicit waters. Thus, while Na^+^ could permeate through the pore with bound BTX^+^, this model cannot explain how BTX could affect the ion selectivity of the channel. It should be noted that at high concentrations the quaternary analog of BTX behaves as the agonist [35].

In the model with BTX^+^/Lys^+^, Na^+^ avoids any contacts with BTX^+^ (Figure 7C) and the BTX^+^: Na^+^ energy is positive all along the pore (Figure 7D). Energy of Na^+^ is also positive at steps 1–16, and slightly negative at some other steps, suggesting that even when the activation gate is open, Na^+^ would not permeate through the channel. An interesting feature of this model is the Na^+^ energy minimum at steps 2–3, although the energy is still positive (Figure 7D). At these steps, Na^+^ distanced from Lys^+^ (Figure 7C), but did not establish favorable contacts with the channel residues. Another feature of this model is the energy of BTX^+^, which is higher than in models with BTX^0^/Lys^0^ (Figure 2 and Figure 3).

### 2.7. Calcium in Model with Lys^0^/BTX^0^

**Figure 8:**
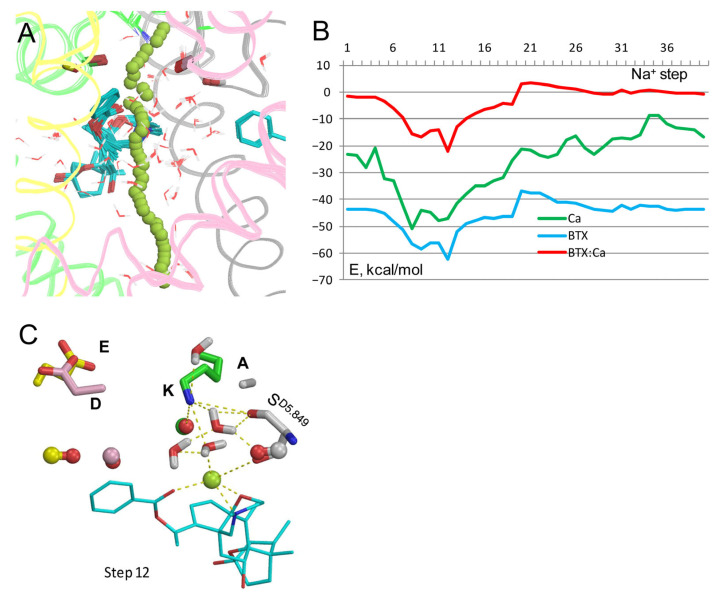
Ca^2+^ in model Lys^0^/BTX^0^. (**A**) Ca^2+^ trajectory. (**B**) Energy profiles from (**A**). The ion experiences a strong attraction to BTX^0^ at step 12. (**C**) Step 12, where Ca^2+^ is at the same level of the pore as the water molecule in the cryoEM structure 8t6l. Ca^2+^ is within 3 Å from three polar atoms of BTX^0^ and a water molecule that is also attracted by Lys^0^. The structure is stabilized by a network of H-bonds involving Lys^0^, S^D5.849^, and four water molecules.

Trajectory of Ca^2+^ (Figure 8A,B) is generally similar to those of Na^+^ in analogous models (Figure 2 and Figure 3). The energy of BTX^0^: Ca^2+^ interaction has a deep minimum of −22 kcal/mol at step 12 and a maximum of 3.3 kcal/mol at step 21. Energy of Ca^2+^ is negative all along the pore, with a wide minimum at steps 4–20. These features are due to strong electrostatic interaction of the divalent cation with oxygen atoms in the outer pore, polar atoms of BTX^0^, water molecules, and macrodipole of helix P1_III_. At step 12, Ca^2+^ interacts with three electronegative atoms of BTX^0^, the backbone carbonyl of S^D5.849^, and a water molecule (Figure 8C). The dunked conformation of Lys^0^ is stabilized by contacts with backbone carbonyls and water molecules. One of the water molecules is also bound to the Ca^2+^ ion, implying its role in stabilizing dunked Lys^0^.

### 2.8. Lys^0^/BTX^0^ in the rNav1.5 Model with the Open Pore Domain

**Figure 9:**
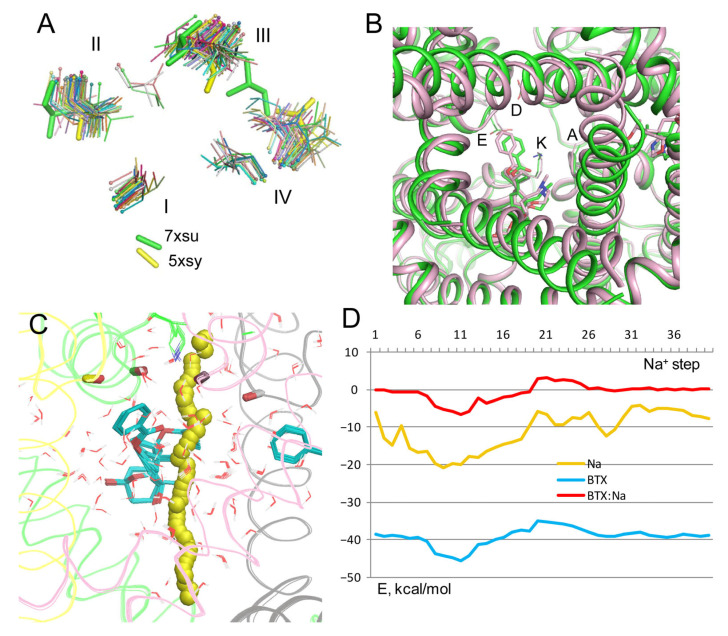
BTX^0^ and Na^+^ in open channel model with Lys^0^. (**A**) Cytoplasmic view of hydrophobic residues 6.553 in 58 cryoEM structures 3D aligned in the PLIC database. The pore lumen in structures of Nav1.4 from electric eel (5xsy) and rNav1.5 with the gating-modified toxin LqhIII (7xsu) is wider than in other structures. (**B**) Cytoplasmic view of the original structure 8tl6 with the inactivated pore domain (magenta) and in the model where the pore domain was in silico opened (green) towards cryoEM structure 7xsu. BTX molecules and DEKA residues underwent rather small displacements upon the pore opening. (**C**,**D**) Na^+^ trajectory in the open pore model is rather similar to that in the inactivated channel (Figure 2).

The activation gate is closed in the majority of 58 cryoEM structures of eukaryotic sodium channels, which are captured with the inactivated pore domain (Figure 9A). Outliers are structures 7xsu and 5xsu, where the pore lumen between hydrophobic residues in positions 6.563 (Table 1) at the cytoplasmic end of the pore is wider than in other structures. Structure 7xsu represents the rNav1.5 channel with the gating modifying toxin LqhIII. To explore how a Na^+^ ion may permeate in this structure, BTX-bound structure 8t6l was in-silico opened. This was performed by MC-minimization of the flexible BTX-bound model in which all C^α^ atoms were pinned to the matching C^α^ atoms in the rigid template 7xsu.

In the resulting structure of BTX-bound open channel, the inner pore is significantly wider than in the 8t6l structure, but the BTX molecules and the DEKA residues underwent relatively small displacements (Figure 9B). The Na^+^ trajectory in the in silico opened model (Figure 9C) and energy profiles of Na^+^, BTX^0^, and BTX^0^: Na^+^ interactions (Figure 9D) are rather similar to respective characteristics in the inactivated channel (Figure 2). This result is consistent with the fact that the BTX molecules bind rather far from the closed activation gate in the 8tl6 structure.

## 3. Discussion

The cryoEM structure 8t6l demonstrated for the first time the binding sites and binding modes of two BTX molecules in the sodium channel [4]. However, the atomic mechanism of BTX action is incompletely understood. In particular, the protonated states of the DEKA lysine and BTX, which should affect the Na^+^ permeation, are unknown. The current study shows that in the models with deprotonated Lys^0^ and BTX^0^, the energy barrier for Na^+^ at the entrance to the inner pore is much lower than in other models. Furthermore, the BTX^0^-attracted Na^+^ ion stabilized the dunked Lys^0^ and thus would affect the ion selectivity. In their break-through study, William Catterall and coworkers did not mention the protonated state of Lys or BTX, but suggested that BTX makes water-mediated hydrogen-bonding interaction with Lys that affects the channel ion selectivity [4]. However, in the cryoEM structure 8t6l with resolution of 3.3 Å, it is hardly impossible to distinguish a water molecule from a Na^+^ ion [31]. In the model with explicit water molecule from structure 8t6l, the permeant Na^+^ ion displaced the water molecule, implying that regardless of what the electron density between BTX and Lys is representing, a Na^+^ ion at this position would stabilize the dunked Lys^0^ and thus affect the ion selectivity (Figure 2C and Figure 3).

The protonation state of the DEKA lysine, which is critical for Na^+^ permeation and selectivity, remains a matter of controversy [13,14]. MD simulations predict that Na^+^ ions have a higher probability to permeate through the outer pore in models with Lys^+^ than in models with Lys^0^ [26,27,28]. In contrast, a MCM-based study suggests that during the permeation cycle, Lys alternates between the protonated and unprotonated states [29]. In fact, both approaches imply that Na^+^ can leave the outer pore when the net electrical charge at the DEKA locus equals zero. In MD-based studies, the net charge of −1 e.u. at the DEKA locus (Asp^−^, Glu^−^, and Lys^+^) is compensated by a Na^+^ ion, but when Lys is deprotonated, the Na^+^ ion remains in the outer pore. In the MCM-based study, the net charge at the DEKA locus is also −1 e.u. (Asp^−^, Glu^−^, Lys^0^, and H^+^) because the proton (H^+^) displaced by Na^+^ from Lys^+^ is proposed to remain nearby until the Na^+^ ion leaves the outer pore [29]. Then, Lys^0^ is reprotonated and Lys^+^ uplifts to Glu^−1^ to complete the permeation cycle. It is the Na^+^ ion chelation between Glu^−1^ and the lone electron pair of Lys^0^ that may explain the ion selectivity of sodium channels [29]. Alternation of a lysine residue between protonated and unprotonated states is proposed, e.g., in the crystallographic study of an amino acid transporter [36].

There are many crystal structures with a Na^+^ ion bound to the lone pair of unprotonated nitrogen. Examples include metal chelators [37,38], structures #137846 and #602323 from the Cambridge Structural Database [39], and a complex with a Na^+^ ion penta-coordinated by two lone pairs of nitrogen atoms, two carbonyl oxygens, and a water molecule [40]. Thus, while at physiological pH, solvent-exposed lysine residues are largely protonated; within the outer pore, the possibility of Na^+^ binding to Lys^0^ cannot be ruled out. A model with Lys^0^ was considered in a recent MD study [26], but Na^+^ was stacked in the outer pore, likely due to electrostatic attraction to Asp^−^ and Glu^−^.

A study of the Na^+^/H^+^ antiporter NhaA has proposed that closely spaced Asp^164^ and Lys^300^ are the key residues involved in the Na^+^/H^+^ exchange; protonated Lys^300^ is salt-bridged to Asp^163^ but a Na^+^ ion may displace the proton and bind between the Asp^−^ and Lys^0^ [41]. Another example of competition between H^+^ and Na^+^ for the tertiary amine is a chelating resin, Chelex 100 [42]. The ratio of Na^+^-bound and protonated nitrogen atoms obviously depends on the local pH. For a buried lysine, pKa may be 3–4 pH units below that for a solvent-exposed lysine [43]. Local pH in the outer pore is unknown, but experimental data indicate that its variation affects the ion permeation. Thus, the effective pK value for proton block of BTX-modified sodium channels in the frog node of Ranvier decreased by ~ 0.4, i.e., shifted to a higher proton concentration [44]. This observation is consistent with the model where BTX and/or Lys are unprotonated at physiological pH, but become protonated at lower pH and thus block the permeation. Another study also demonstrated that reduction in the extracellular pH inhibits Na^+^ permeation through sodium channels [45].

**Figure 10:**
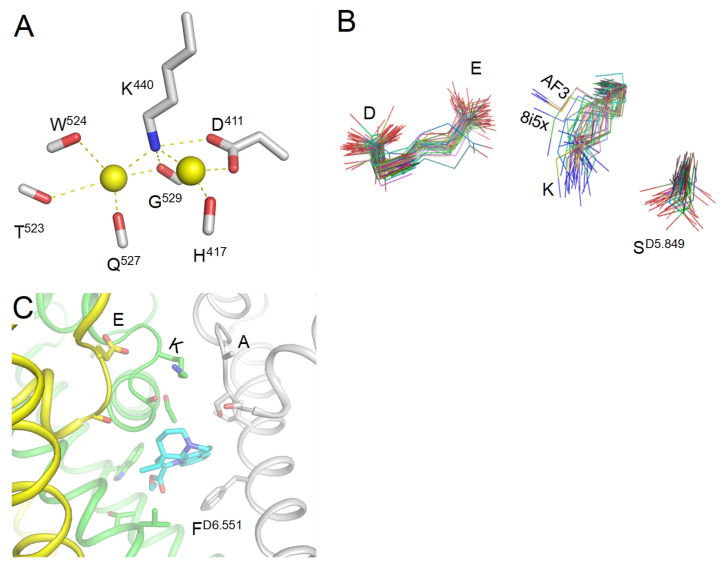
Lysine in experimental structures. (**A**) In a high-resolution X-ray structure of aminopeptidase [46], two sodium ions bind to a lysine residue (PDB ID: 6ryf). One sodium ion is chelated between the lysine and aspartate, like in the proposed model where a sodium ion displaces a proton from the uplifted Lys^+^ and binds between Lys^0^ and Glu^−^ [29]. (**B**) Sidechains of the DEKA residues in 58 cryoEM structures and four AF3 models of Nav1.x channels. The sidechains are extracted from the PLIC database. In the majority of cryoEM structures, the DEKA lysine is dunked, but note outlier 8i5x. In the AlphaFold3 (AF3) models, this lysine is lifted, approaching the DEKA glutamate. Noted different orientation of the S^D5.849^ sidechain that in some cryoEM structures is H-bonded to the lysine. (**C**) CryoEM structure of the Nav1.7 channel with vinprocetine at resolution 2.9 Å (8i5x). The uplifted sidechain of the DEKA lysine approaches the DEKA glutamate. Repeat A is removed for clarity.

An important evidence that deprotonated lysine may interact with a Na^+^ ion is seen in the high-resolution X-ray structure of aminopeptidase from endoplasmic reticulum (6ryf) where two Na^+^ ions bind to a lysine residue [46]. One Na^+^ ion is chelated between the lysine and an aspartate (Figure 10A), similarly to the model where a Na^+^ ion displaces a proton from the uplifted Lys^+^ and binds between Lys^0^ and Glu^−^ [29].

The majority of cryoEM structures of Nav1.x channels show dunked DEKA Lys, but in AlphaFold models, the uplifted sidechain of Lys approaches Glu (Figure 10B). In the CryoEM structure of the Nav1.7 channel with vinprocetine (8i5x) obtained at resolution of 2.9 Å [47], the uplifted sidechain of Lys approaches Glu (Figure 10C), and the distance of 4.5 Å between the Lys nitrogen and a Glu oxygen is appropriate to chelate a water molecule or a Na^+^ ion.

The latter structure further supports the following proposition on how BTX may increase permeation of Ca^2+^ ions. A Na^+^ ion passing through the pore encounters the least resistance in the model with BTX^0^/Lys^0^ (Figure 2 and Figure 3). And although Na^+^ could permeate in the model with Lys^0^/BTX^+^, it bypassed BTX (Figure 7A,B) without stabilizing the dunked Lys^0^. In models with Lys^0^/BTX^0^, BTX^0^-bound Na^+^ or Ca^2+^ ions stabilized the dunked Lys^0^, which is attracted by the permeant cation, nearby water molecules, backbone carbonyls, and the sidechain of S^D5.848^ (Figure 2C,D, Figure 3C and Figure 8C). This attraction would retard reprotonation of Lys^0^ and its uplifting to Glu^−^ [29]. When uplifting of Lys^+^ is delayed, Asp^−^ and Glu^−^ would attract a Ca^2+^ ion.

The fact that BTX reduces single-channel conductance [12] is consistent with the partial pore occlusion by the toxin (Figure 1) and predicted energy barriers for Na^+^ around step 20 (Figure 2B and Figure 3B), which is higher than in the apo-channel (Figure 4B). BTX-induced hyperpolarizing shifts in the voltage-dependence of activation and fast inactivation are proposed to be due to BTX stabilization of the π-helical conformation of S6_I_ and S6_III_ and α-helical conformation of S6_II_ and S6_IV_ [4].

## 4. Conclusions

Among models with different protonation states of the DEKA lysine and BTX explored in this study, only in the model with Lys^0^/BTX^0^, Na^+^ and Ca^2+^ ions readily moved from the outer pore through the inner pore and experienced attraction to BTX^0^. This is understandable given strong electrostatic interactions in the low-dielectric environment within a membrane protein. The current study suggests how BTX may cause a manifold increase in Ca^2+^ permeation through the Na^+^ channel [8]. Both Na^+^ and Ca^2+^ ions were attracted to the pore-exposed electronegative atoms of the BTX^0^ molecule in the III/IV repeat interface, and stabilized the dunked conformation of Lys^0^ through electrostatic interactions and nearby water molecules. These interactions decrease the probability of the DEKA lysine reprotonation and its uplifting to the DEKA Glu^−1^. When the DEKA lysine is retarded in the dunked conformation, the probability of the Ca^2+^ attraction by the two acidic DEKA residues is increased. The Ca^2+^ ion would then move through the pore as long as Lys and BTX are unprotonated.

## 5. Materials and Methods

The all-atom AMBER force field [48] was used to calculate nonbonded interactions. Parameters for Na^+^ and Ca^2+^ were from AMBER-9. Atomic charges in BTX-B were calculated by MOPAC [49]. Electrostatic interactions were calculated with the dielectric function *ε* = *2d*, where *d* is distance between two atoms. No distance cutoff was used to calculate electrostatic interactions involving Na^+^, Ca^2+^, or ionized groups in BTX and amino acids. For other interactions, the distance cutoff of 9 Å and a shifting function [50] were used. Models were Monte Carlo (MC)-energy-minimized [51] using the ZMM program (www.zmmsoft.ca), accessed on 22 September 2025, that optimizes energy in the space of internal (generalized) coordinates [52]. These include all torsional angles, positions, and orientations (Euler angles) of BTX and water molecules, coordinates of the permeant ions, and bond angles in the toxin molecules and proline residues.

MC-minimized trajectories of Na^+^ and Ca^2+^ ions were computed as follows. An ion progressively moved through 40 planes, which are normal to the pore axis. The planes are also referred to as pore levels or steps. Two adjacent planes are 0.5 Å apart. The *z*-coordinate of the first plane is −5.0 Å. For comparison, the *z*-coordinate of the pore-facing backbone oxygen in T^C5.848^ is −7.5 Å, i.e., 2.5 Å below the first plane. This level of the first plane is chosen to explore the ion permeation from the outer pore exit and within the BTX-bound inner pore. Trajectories of Na^+^ in the outer pore have been explored before, using the same methodology as in the current study [29]. At each step, the ion was allowed to move within the plane, but not to leave it. At each step, the MCM trajectory was terminated when the last 500 consecutive energy minimizations did not improve the apparent global minimum found at the step. At a given step, starting positions of the permeant ion and BTX, as well as all torsional angles in the channel and BTX did not depend on characteristics obtained at the previous step. This ruled out a possibility that the lowest energy ion position at any step would influence the ion position at the next step.

To prevent large structural deformations due to possible clashes of residues during MC-minimizations, C^α^ atoms of the protein were constrained by pins. A pin is a distance constraint between the experimental and computed positions of a C^α^ atom that allows its energy-free deviations up to 1 Å from the experimental position and imposes an energy penalty using a parabolic function with the force constant of 10 kcal mol^−1^ Å^−1^. Such pins were also used to bias experimental positions of heavy atoms in the BTX molecules and the DEKA lysine.

An equilibrated sphere of 500 water molecules [53] was used to hydrate the pore. The sphere of ~30 Å in diameter obtained from Gromacs [54] was positioned to wet all the pore-lining residues between the outer carboxylates and the activation gate. Such wetting was sufficient given that the length of an ion trajectory along the pore axis is 20 Å. In the beginning of each energy minimization, a model was completely rewetted and water molecules closer than 2 Å from a heavy atom in the channel, in BTX, or to the permeant ion, were removed. This ensured that an MC-sampled structure with a large displacement of the permeant ion or with a significant change in a sidechain conformation had a chance to be accepted into the MCM trajectory. For the sake of comparison, a model with BTX^0^/Lys^0^ was also explored without explicit waters, by using the environment- and distance-dependent dielectric function for electrostatic interactions [33] or implicit solvent [34].

Molecular images were created using the PyMol Molecular Graphics System, Version 0.99rc6 (Schrödinger, LLC, New York, NY, USA).

## Figures and Tables

**Table 1:** UniProt numbers and PLIC labels of residues in helices S4–S5, S5, P1, P2, and S6 of rNav1.5. The sequences are shown according to cryoEM structure 8t6l.

rNav1.5	Repeat	======= S4–S5 ===== ======================== S5 ====================			
		5.518	5.535	5.540	5.550
233	A	ISGLKTIVGALIQSVKK	LADVM	VLTVFCLSVF	**A**LIGLQLF
823	B	WPTLNTLIKIIGNSVGA	LGNLT	LVLAIIVFIF	**A**VVGMQLF
1319	C	FEGMRVVVNALVGAIPS	IMNVL	LVCLIFWLIF	**S**IMGVNLF
1642	D	AKGIRTLLFALMMSLPA	LFNIG	LLLFLVMFIY	**S**IFGMANF
		**======== P1 ========~~~== P2 ===========**			
		5.836	5.850		
359	A	FAWAFLALFRLMTQ	**D**CWERLYQQ		
887	B	FFHAFLIIFRILCG	**E.**WIETMWD		
1407	C	VGAGYLALLQVATF	**K**GWMDIMYA		
1699	D	FANSMLCLFQITTS	**A**GWDGLLSP		
		**============================= S6 =====================================**			
		6.537	6.550	6.560	6.570
388	A	KIYMIFFMLVIFL	**G**SFYLVNLIL	AVVAMAYEEQ	NQATIAETEEKEKR
916	B	SLCLLVFLLVMVI	**G**NLVVLNLFL	ALLLSSF	
1446	C	LYMYIYFVVFIIF	**G**SFFTLNLFI	GVIIDNFNQQ	KKKL
1748	D	AVGILFFTTYIII	**S**FLIVVNMY	IAIILENFSVA	

## Data Availability

The original contributions presented in this study are included in the article. Further inquiries can be directed to the corresponding author.
